# Gravitational form factors of the Higgs boson

**DOI:** 10.1140/epjc/s10052-025-15139-0

**Published:** 2025-12-27

**Authors:** P. Beißner, B.-D. Sun, E. Epelbaum, J. Gegelia

**Affiliations:** 1 Institut für Theoretische Physik II, Ruhr-Universität Bochum, 44780 Bochum, Germany; 2 Tbilisi State University, 0186 Tbilisi, Georgia

## Abstract

We calculate the one-loop electroweak corrections to the gravitational form factors of the Higgs boson and discuss the interpretation of the obtained results.

## Introduction

After 100 years of quantum mechanics we still continue to use the language of the classical physics to describe subatomic systems. While we certainly do not think that these are in any sense classical objects we still operate with terms like, e.g., charge radius, meaning that in low-energy electromagnetic scattering experiments in first approximation they behave like as if subatomic systems were classical objects with charge distributions and the corresponding mean-square radii.

Being tempted to adopt (a rather problematic) definition of elementary particles as those which are represented by own local field operators in the standard model Lagrangian [1] one may ask if these particles are point-like. This question cannot be answered unless one specifies the precise meaning of the term point-like. Probing subatomic systems via electromagnetic interactions, electrons behave like as if they had pointlike charge-distribution if one stays at leading order in the expansion in powers of the electromagnetic coupling. Protons, on the other hand, possess non-trivial electromagnetic form factors and therefore are seen as extended objects. This picture of nucleons occurs due to the strong interaction, which is much stronger than the electromagnetic one and needs to be taken into account also when the electromagnetic interaction is treated only at tree order, i.e., within the classical approximation. Similarly to the electromagnetic case, one can, at least in principle, also probe subatomic systems with gravitons. While it is not feasible to measure scattering processes of such systems off external gravitational sources, theoretical investigation can be carried out in the framework of the standard model of particle physics. Such scattering processes in the single-graviton approximation are described by diagrams in which the system under consideration couples to the energy-momentum tensor (EMT). Matrix elements of the EMT and the corresponding gravitational form factors (GFF) [2, 3] have been extensively studied for various systems in recent years. In particular, the GFFs of hadrons have attracted much attention, see, e.g.,Refs. [4–27]. Calculations of gravitational structure due to strong interaction effects have been performed using different methods. The increased interest to this topic is driven by the fact that GFFs of hadrons can be (indirectly) extracted from experiment. Compared to the gravitational force, electromagnetic and weak interactions are very strong and, therefore, it makes sense to consider also the electroweak corrections to matrix elements of the EMT. Corrections due to the electromagnetic interaction lead to infrared divergences [28–31] that require taking into account emission of soft photons. In this work, we calculate the one-loop electroweak corrections to the matrix element of the EMT operator for the Higgs boson, which is electrically neutral, and provide interpretation of the obtained results in terms of spatial densities defined via sharply localized states [32].

Our paper is organized as follows: In Sect. 2 we specify some details of the electroweak theory, which are needed for our calculation. Next, we calculate in Sect. 3 the matrix element of the EMT operator for the Higgs boson and extract GFFs. The results of our work are summarized in Sect. 4.

## Lagrangian and the energy-momentum tensor

In our calculations we use the Lagrangian and the Feynman rules of the electroweak theory as specified in Ref. [33]. The EMT operator of a non-Abelian gauge theory with spontaneous symmetry breaking can be found in Ref. [34]. We exploited the results of this work to obtain the EMT operator needed for our calculations by considering the Lagrangian of the electroweak theory in the presence of the external gravitational field and using the definition [35]

1 $$\begin{aligned} T_{\mu \nu } (g,\psi )= &   \frac{2}{\sqrt{-g}}\frac{\delta S_\textrm{m} }{\delta g^{\mu \nu }} \end{aligned}$$

for matter fields interacting with the gravitational metric fields. For the fermion fields interacting with gravitational vielbein fields, we employ the definition [35]

2 $$\begin{aligned} T_{\mu \nu } (g,\psi )= &   \frac{1}{2 e} \left[ \frac{\delta S }{\delta e^{a \mu }} \,e^{a}_\nu + \frac{\delta S }{\delta e^{a \nu }} \,e^{a}_\mu \right] , \end{aligned}$$

where *e* is the determinant of $e^a_\mu$.

In the calculation of one-loop diagrams below we apply dimensional regularization (see, e.g., Ref. [36]) with *D* spacetime dimensions and use the program FeynCalc [37–39]. The results of our calculations are expressed in terms of scalar integrals defined in Appendix A.

## One-loop corrections to the gravitational form factors

Using the conventions of Ref. [40], we parameterize the one-particle matrix element of the EMT operator for a scalar particle as

3 $$\begin{aligned} \langle p' | T_{\mu \nu }| p\rangle  &   = \frac{1}{2} \left( g_{\mu \nu } q^2-q_\mu q_\nu \right) \theta _1(q^2)\nonumber \\ \quad  &   + \frac{1}{2} \, P_\mu P_\nu \, \theta _2(q^2) , \end{aligned}$$

where $q=p'-p$ and $P=p+p'$. The gravitational form factor $\theta _2(q^2)$ satisfies the normalization condition $\theta _2(0)=1$, while $\theta _1(q^2)$ gives rise to the D-term $D= - \theta _1(0)$, which is not constrained by normalization.

**Fig. 1:**
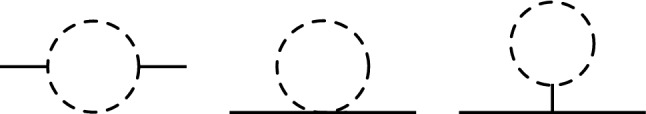
Topologies of one-loop diagrams contributing to the self-energy of the Higgs boson. Solid lines correspond to Higgs bosons and the dashed lines represent vector bosons, fermions, Higgs bosons, Goldstone bosons and Faddeev–Popov ghosts

We obtain the Higgs boson matrix element of the EMT operator by applying the standard LSZ formalism to the vacuum expectation value of the time-ordered product of the EMT operator and two Higgs boson fields. To do so at one-loop order we first need to calculate the one-loop contributions to the pole position and the residue of the dressed propagator of the Higgs boson given by

4 $$\begin{aligned}  &   S(p) \; =\; \frac{i}{p^2-M_{H}^2+ \Sigma } =\frac{i \, Z}{p^2- z} \; + \; \text {non-pole part} , \end{aligned}$$

where $M_H$ is the mass of the Higgs boson, $\Sigma$ is its self-energy, $z= M_H^2 + \sum _{j=1}^{\infty } \hbar ^j \delta z_j$ denotes the pole position of the dressed propagator while $Z=1+\sum _{j=1}^{\infty } \hbar ^j \delta Z_j$ is the residue at the pole. Here, the series in terms of $\hbar$ correspond to the loop expansion. Topologies of one-loop diagrams contributing to the self-energy of the Higgs boson are shown in Fig. 1. The corresponding expressions for the one-loop contributions to *z* and *Z* are given in appendix B.

**Fig. 2:**
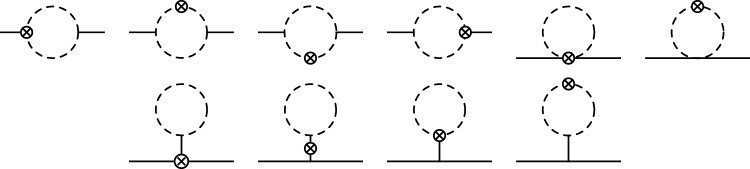
Topologies of one-loop diagrams contributing to the three-point vertex function of the EMT operator and two Higgs boson fields. The crosses stand for the EMT insertions, solid lines refer to Higgs bosons, while dashed lines represent vector bosons, fermions, Higgs bosons, Goldstone bosons and Faddeev–Popov ghosts

The one-loop topologies contributing to the three-point function are shown in Fig. 2. By calculating these diagrams and subsequently applying the LSZ formalism we extract the GFFs, whose explicit expressions are given in appendix C. We find that $\theta _2 (q^2)$ is ultraviolet finite while $\theta _1 (q^2)$ diverges, and the corresponding divergence cannot be eliminated by renormalization of the parameters in the electroweak Lagrangian, in agreement with the results of Refs. [34, 41, 42]. The (momentum-independent) divergent part of $\theta _1(q^2)$ is given by

5 $$\begin{aligned} \theta _1^\textrm{div}= \frac{e^2 M_Z^2 \left( 3 M_H^2+2 m_n^2-6 M_W^2-3 M_Z^2\right) }{24 \pi ^2 (D-4) M_W^2\left( M_Z^2-M_W^2\right) } , \end{aligned}$$

where $M_Z$, $M_W$ and $m_n$ are the masses of the *Z* and *W* vector bosons, and *n*-th fermion, respectively, while *e* is the electric charge. This divergence is canceled by the counter term generated by the corresponding term of the Lagrangian $\sim R \phi ^2$, where *R* is the scalar curvature [42]. Such term with a free parameter appears in the EFT Lagrangian that contains all local interactions compatible with the symmetries of the electroweak theory and the general coordinate transformations. Notice that the “improvement term” of the energy-momentum tensor introduced in Ref. [41] corresponds to the term $\sim R \phi ^2$ of the effective Lagrangian, however with a fixed numerical value of the corresponding coupling constant.

By considering localized one-particle states, the GFFs can be related to the corresponding spatial distributions [32]. However, physical interpretation of the latter poses certain challenges. Problem occurs due to the fact that a superposition of eigenstates of the energy-momentum four-vector with different eigenvalues, which forms a localized state of the particle, is not an eigenstate of this operator. However, if the non-relativistic approximation is valid, the states can be defined by wave packets with the size much larger than the Compton wavelength of the system (and much smaller than characteristic radii of the system). For such wave packets, the integral over momentum eigenstates is governed by momenta much smaller than the mass of the system. Therefore, replacing the corresponding energies by the first term in the expansion $E=\sqrt{m^2+\textbf{p}^2}=m+\textbf{p}^2/(2 m)+\cdots$ provides a good approximation. That is, the packet is dominated by eigenstates of the energy with the same eigenvalue *m*, and therefore it is also an (approximate) eigenstate of the energy operator with the eigenvalue *m*. Thus, $t^{00} (\textbf{r})$ can be interpreted in this case as the spatial distribution of mass, which in the static approximation coincides with the energy distribution of the system. More details of the interpretation of the Fourier transforms of the gravitational form factors in the Breit frame in terms of various spatial distributions can be found in Ref. [6].

In the case of the Higgs boson considered here, the static approximation is by no means applicable as the slopes of the form factors at zero momentum transfer, $d \theta _i /dq^2 \big |_{q^2 = 0}$, which give rise to the characteristic size of the system, are smaller than the Compton wavelength squared of the Higgs boson. Therefore, we need to consider sharply localized states with the size *R* of the wave packet $\phi$ chosen much smaller than the Compton wave length. Such wave packet states are dominated by high momenta, and the spatial distribution given by [32]

6 $$\begin{aligned}  &   t^{00}(\textbf{r}) =N_{\phi ,R} \, \int \frac{d^2 \hat{n} \,d^3 {q}}{(2\pi )^3} \, \theta _2 ( -\textbf{q}_\perp ^2 ) \, e^{-i \textbf{q}\cdot \textbf{r}} \end{aligned}$$

can be interpreted as energy distribution. Here, $\textbf{q}_\perp = \textbf{q} - \textbf{q} \cdot \hat{\textbf{n}} \hat{\textbf{n}}$. Notice that while the overall normalization $N_{\phi ,R}$ depends on the form of the packet and diverges when the packet size is reduced, the shape of the distribution does not depend on the details of the (spherically symmetric) wave packet used to prepare the system. Our interpretation is that the normalization $N_{\phi ,R}$ diverges in the limit $R \rightarrow 0$ because one needs an increasing amount of energy to reduce the size of the packet, while the shape of the distribution is uniquely determined by the corresponding form factor and characterizes the internal energy distribution of the system.

The energy distribution of Eq. (6) corresponding to sharply localized states with the size *R* of the wave packet $\phi$ much smaller than the Compton wave length, leads to the three-dimensional mean-square energy-radius

7 $$\begin{aligned}  &   r^2 = 4 \frac{d \theta _2 ( q^2 )}{dq^2} \bigg |_{q^2 = 0} . \end{aligned}$$

From our result for $\theta _2(t )$ we obtain

8 $$\begin{aligned}  &   r^2 = -\frac{e^2 \left( 6 m_n^2+M_H^2\right) M_Z^2 B_0\left( M_H^2,m_n^2,m_n^2\right) m_n^4}{24 \pi ^2 M_H^4 \left( M_H^2-4 m_n^2\right) M_W^2 \left( M_W^2-M_Z^2\right) }\nonumber \\  &   \quad +\frac{e^2 \left( 6m_n^2+M_H^2\right) M_Z^2 A_0\left( m_n^2\right) m_n^2}{24 \pi ^2 M_H^4 \left( M_H^2-4 m_n^2\right) M_W^2 \left( M_W^2-M_Z^2\right) } \nonumber \\  &   \quad + \frac{e^2 M_Z^2 A_0\left( M_H^2\right) }{24 \pi ^2 M_H^2 M_W^2 \left( M_W^2-M_Z^2\right) }\nonumber \\  &   \quad +\frac{e^2 \left( 5 M_H^6-29M_W^2 M_H^4+24 M_W^4 M_H^2+36 M_W^6\right) M_Z^2 A_0\left( M_W^2\right) }{24 \pi ^2 M_H^4 M_W^2 \left( M_H^2-4 M_W^2\right) {}^2 \left( M_W^2-M_Z^2\right) } \nonumber \\  &   \quad + \frac{e^2 M_Z^2 \left( 5 M_H^6-29 M_Z^2 M_H^4+24 M_Z^4 M_H^2+36 M_Z^6\right) A_0\left( M_Z^2\right) }{48 \pi ^2 M_H^4 M_W^2 \left( M_H^2-4 M_Z^2\right) {}^2 \left( M_W^2-M_Z^2\right) }\nonumber \\  &   \quad -\frac{e^2 M_Z^2 B_0\left( M_H^2,M_H^2,M_H^2\right) }{24 \pi ^2 M_W^2 \left( M_W^2-M_Z^2\right) } \nonumber \\  &   \quad - \frac{e^2 \left( 5 M_H^6-29 M_W^2 M_H^4+24 M_W^4 M_H^2+36 M_W^6\right) M_Z^2 B_0\left( M_H^2,M_W^2,M_W^2\right) }{24 \pi ^2 M_H^4 \left( M_H^2-4 M_W^2\right) {}^2 \left( M_W^2-M_Z^2\right) } \nonumber \\  &   \quad - \frac{e^2 M_Z^4 \left( 5 M_H^6-29 M_Z^2 M_H^4+24 M_Z^4 M_H^2+36 M_Z^6\right) B_0\left( M_H^2,M_Z^2,M_Z^2\right) }{48 \pi ^2 M_H^4 M_W^2 \left( M_H^2-4 M_Z^2\right) {}^2 \left( M_W^2-M_Z^2\right) }\nonumber \\  &   \quad +\frac{e^2 \left( M_H^6-24 m_n^6\right) M_Z^2}{96 \pi ^2 M_H^4 \left( M_H^2-4 m_n^2\right) M_W^2 \left( M_W^2-M_Z^2\right) } \nonumber \\  &   \quad - \frac{e^2 M_Z^2 }{96 \pi ^2 M_H^4 M_W^2 \left( M_H^2-4 M_W^2\right) {}^2 \left( M_H^2-4 M_Z^2\right) {}^2 \left( M_W^2-M_Z^2\right) } \nonumber \\  &   \quad \times \Bigl [ 144 \left( M_H^2-4 M_Z^2\right) {}^2 M_W^8 + 72 \left( M_H^3-4 M_H M_Z^2\right) {}^2 M_W^6\nonumber \\  &   \quad +16 \left( 5 M_H^8-41 M_Z^2 M_H^6+72 M_Z^4 M_H^4+36 M_Z^6 M_H^2+72 M_Z^8\right) M_W^4 \nonumber \\  &   \quad - 2 M_H^2 \left( 25 M_H^8-204 M_Z^2 M_H^6+368 M_Z^4 M_H^4+144 M_Z^6 M_H^2+288 M_Z^8\right) M_W^2\nonumber \\    &   \quad +M_H^4 \left( 6 M_H^8-49 M_Z^2 M_H^6 \right. \nonumber \\  &   \quad + \left. 88 M_Z^4 M_H^4+36 M_Z^6 M_H^2+72 M_Z^8\right) \Bigr ]. \end{aligned}$$

This expression is ultraviolet finite and its value in four spacetime dimensions does not depend on the scale parameter of dimensional regularization. Substituting numerical values of various parameters from Ref. [43] we estimate:

9 $$\begin{aligned} r^2 = 5.61\times 10^{-8} + (2.14\times 10^{-9} + 4.3\times 10^{-14} \, i) \ \textrm{fm}^2\,, \end{aligned}$$

where the number in the brackets refers to the contribution of fermions. Notice here that unstable particles have in general complex form factors leading to complex-valued static properties, see, e.g., Refs. [44–46]. The expression of Eq. (9) also contains an imaginary part as the Higgs particle is unstable, decaying in fermion-anti-fermion pairs.

Our expression of the one-loop correction to the D-term of the Higgs boson has the form:

10 $$\begin{aligned}  &   D = -\frac{e^2 m_n^2 M_Z^2 B_0\left( M_H^2,m_n^2,m_n^2\right) }{48 \pi ^2 M_W^2 \left( M_W^2-M_Z^2\right) } \nonumber \\  &   \quad -\frac{e^2 M_H^2 M_Z^2 B_0\left( M_H^2,M_H^2,M_H^2\right) }{32 \pi ^2 M_W^2 \left( M_W^2-M_Z^2\right) } \nonumber \\  &   \quad + \frac{e^2 M_Z^2 A_0\left( m_n^2\right) }{16 \pi ^2 M_W^2 \left( M_W^2-M_Z^2\right) } \nonumber \\  &   \quad + \frac{e^2 M_Z^2 \left( 4 M_H^2 M_W^2-M_H^4+12 M_W^4\right) B_0\left( M_H^2,M_W^2,M_W^2\right) }{48 \pi ^2 M_W^2 \left( 4 M_W^2-M_H^2\right) \left( M_W^2-M_Z^2\right) }\nonumber \\  &   \quad +\frac{e^2 M_Z^2 \left( 4 M_H^2 M_Z^2-M_H^4+12 M_Z^4\right) B_0\left( M_H^2,M_Z^2,M_Z^2\right) }{96 \pi ^2 M_W^2 \left( 4 M_Z^2-M_H^2\right) \left( M_W^2-M_Z^2\right) } \nonumber \\  &   \quad + \frac{e^2 M_Z^2 A_0\left( M_H^2\right) }{16 \pi ^2 M_W^2 \left( M_W^2-M_Z^2\right) }\nonumber \\  &   \quad -\frac{e^2 M_Z^2 A_0\left( M_W^2\right) \left( 6 M_W^2-M_H^2\right) }{8 \pi ^2 M_W^2 \left( 4 M_W^2-M_H^2\right) \left( M_W^2-M_Z^2\right) } \nonumber \\  &   \quad + \frac{e^2 M_Z^2 A_0\left( M_Z^2\right) \left( M_H^2-6 M_Z^2\right) }{16 \pi ^2 M_W^2 \left( 4 M_Z^2-M_H^2\right) \left( M_W^2-M_Z^2\right) }\nonumber \\  &   \quad - \frac{e^2 M_Z^2 }{48 \pi ^2 M_W^2 \left( 4 M_W^2-M_H^2\right) \left( 4 M_Z^2-M_H^2\right) \left( M_W^2-M_Z^2\right) } \nonumber \\  &   \quad \times \Bigl [ -m_n^2 \left( 4 M_W^2-M_H^2\right) \left( M_H^2-4 M_Z^2\right) +4 M_W^4 \left( 4 M_Z^2-M_H^2\right) \nonumber \\  &   \quad + 2 M_W^2 \left( 30 M_H^2 M_Z^2-7 M_H^4+4 M_Z^4\right) \nonumber \\  &   \quad -13 M_H^4 M_Z^2-2 M_H^2 M_Z^4+3 M_H^6 \Bigr ]. \end{aligned}$$

As mentioned above, the *D*-term contains ultraviolet divergence which is cancelled by the counter term generated by the term $$h\, R \phi ^2$$ of the effective Lagrangian. The renormalization scale dependence of the renormalized parameter $$h_R$$ cancels the scale-dependence of the expression of Eq. (10). The value of $$h_R$$ is a free parameter in EFT. On the other hand, by taking its value from the “improvement term” of Ref. [41] one obtains $$D=-1/3 $$ at tree order [47].

## Summary

In the current work we have calculated the electroweak correction to the matrix element of the EMT of the Higgs boson to one loop and extracted the corresponding GFFs. We found that the $\theta _2(q^2)$ GFF is ultraviolet finite, while the $\theta _1 (q^2)$ diverges, in agreement with Refs. [34, 41, 42]. This divergence cannot be cancelled by counter terms generated by the Lagrangian of the electroweak theory minimally coupled to the gravitational field. However the corresponding dimension-four operator is present in the standard-model EFT Lagrangian that contains all local interactions compatible with underlying symmetries [42]. By considering matrix elements of the EMT operator for localized wave packet states, the GFFs can be related to corresponding spatial distributions [32]. Our expressions for both form factors $\theta _1(q^2)$ and $\theta _2 (q^2)$ have non-trivial dependence on the momentum transfer squared. The characteristic radius of the energy distribution of the Higgs boson, defined by the slope of the form factor $\theta _2(q^2)$ at vanishing momentum transfer, is smaller than the Compton wave length of the Higgs boson. Therefore, it is not appropriate to consider the Breit-frame densities corresponding to the static approximation. On the other hand, the gravitational interaction probes the energy distribution of the Higgs boson (and reveals a non-zero energy-radius) through the matrix element of the EMT operator between sharply localized states with the wave packet size chosen to be much smaller than the Compton wavelength of the Higgs boson. Clearly, it is not feasible to measure gravitational scattering off the Higgs boson in experiment, and it is also unfeasible to localize it at distances smaller than its Compton radius. Still, our theoretical investigation demonstrates that an electrically neutral elementary particle reveals internal structure with a non-vanishing spatial extension when being probed by the gravitational interaction. One might speculate that in analogy with charged particles, the non-zero radius emerges due to the weak field of massive vector particles, however we find that similar in size contributions to the radius are generated by diagrams with fermion loops and self-interactions of the scalar field.

## Data Availability

This manuscript has no associated data. [Authors’ comment: Data sharing not applicable to this article as no datasets were generated or analysed during the current study].

## Appendix A Definition of loop integrals

One-loop integrals are defined as follows:

A1 $$\begin{aligned}  &   \text {A}_0(m^2)=\frac{(2\pi \mu )^{4-D}}{i\pi ^2}\int \frac{d^D k}{k^2-m^2+i \epsilon },\nonumber \\  &   \text {B}_0(p^2,m_1^2,m_2^2)=\frac{(2\pi \mu )^{4-D}}{i\pi ^2} \nonumber \\  &   \quad \times \int \frac{d^Dk}{[k^2-m_1^2+i \epsilon ] [(p+k)^2-m_2^2+i \epsilon ]}, \nonumber \\  &   C_0(p_1^2,p_2^2,p^2_{12},m_1^2,m_2^2,m_3^2)=\frac{(2\pi \mu )^{4-n}}{i\pi ^2} \nonumber \\  &   \quad \times \int \frac{d^nk}{[k^2-m_1^2+i \epsilon ] [(p_1+k)^2-m_2^2+i \epsilon ] [(p_1+p_2+k)^2-m_ 3^2+i \epsilon ]}, \end{aligned}$$

where $p_{12}=p_1+p_2$ and *D* is the spacetime dimension and $\mu$ the scale parameter. For tensor loop integrals, we apply the reduction formulae of Ref. [48], while for the expansion of the scalar integrals in Eq. (A1) in terms of kinematical invariants we use Ref. [49].

## Appendix B Contributions to the pole position and the residue of the Higgs propagator

One-loop contributions to the pole position and the residue of the Higgs-boson propagator are given by

B1 $$\begin{aligned} \delta z_1= &   \frac{ - e^2 M_Z^2 }{128 \pi ^2 M_W^2 \left( M_W^2-M_Z^2\right) }\left( \left( -8 (D-1) M_W^4+8 M_H^2 M_W^2-2 M_H^4\right) \right. \nonumber \\  &   \times \text {B}_0\left( M_H^2,M_W^2,M_W^2\right) \nonumber \\  &   + \left. \left( -4 (D-1) M_Z^4+4 M_H^2 M_Z^2-M_H^4\right) \right. \nonumber \\  &   \times \text {B}_0\left( M_H^2,M_Z^2,M_Z^2\right) -4 m_n^2 \left( M_H^2-4 m_n^2\right) \nonumber \\  &   \times \left. \text {B}_0\left( M_H^2,m_n^2,m_n^2\right) - 9 M_H^4 \text {B}_0\left( M_H^2,M_H^2,M_H^2\right) \right. \nonumber \\  &   +4 \text {A}_0\left( M_W^2\right) \left( 2 (D-1) M_W^2+M_H^2\right) \nonumber \\  &   +2 \text {A}_0\left( M_Z^2\right) \left( 2 (D-1) M_Z^2+M_H^2\right) \nonumber \\  &   + \left. 6 M_H^2 \text {A}_0\left( M_H^2\right) -16 m_n^2 \text {A}_0\left( m_n^2\right) \right) ,\nonumber \\ \delta Z_1= &   -\frac{e^2 M_Z^2}{128 \pi ^2 M_W^2 \left( M_W^2-M_Z^2\right) } \nonumber \\  &   \times \Biggl [ -\frac{4 \left( 4 (D-1) M_W^6-5 M_H^4 M_W^2+4 M_H^2 M_W^4+M_H^6\right) \text {B}_0\left( M_H^2,M_W^2,M_W^2\right) }{M_H^4-4 M_H^2 M_W^2} \nonumber \\  &   - \frac{2 \left( 4 (D-1) M_Z^6-5 M_H^4 M_Z^2+4 M_H^2 M_Z^4+M_H^6\right) \text {B}_0\left( M_H^2,M_Z^2,M_Z^2\right) }{M_H^4-4 M_H^2 M_Z^2} \nonumber \\  &   - \left( \frac{8 m_n^4}{M_H^2} + 4 m_n^2\right) \text {B}_0\left( M_H^2,m_n^2,m_n^2\right) \nonumber \\  &   + 4 M_H^2 \text {B}_0\left( M_H^2,M_W^2 \alpha _W,M_W^2 \alpha _W\right) \nonumber \\  &   +2 M_H^2 \text {B}_0\left( M_H^2,M_Z^2 \alpha _Z,M_Z^2 \alpha _Z\right) \nonumber \\  &   +6 M_H^2\text {B}_0\left( M_H^2,M_H^2,M_H^2\right) \nonumber \\  &   + \frac{8 \text {A}_0\left( M_W^2\right) \left( 2 (D-1) M_W^4-4 M_H^2 M_W^2+M_H^4\right) }{M_H^4-4 M_H^2 M_W^2}\nonumber \\  &   +\frac{4 \text {A}_0\left( M_Z^2\right) \left( 2 (D-1) M_Z^4-4 M_H^2 M_Z^2+M_H^4\right) }{M_H^4-4 M_H^2 M_Z^2} \nonumber \\  &   + \frac{8 m_n^2\text {A}_0\left( m_n^2\right) }{M_H^2}-6 \text {A}_0\left( M_H^2\right) \nonumber \\  &   -4\text {A}_0\left( M_W^2 \alpha _W\right) -2 \text {A}_0\left( M_Z^2 \alpha _Z\right) \nonumber \\  &   + \frac{1}{M_H^2 \left( M_H^2-4 M_W^2\right) \left( M_H^2-4 M_Z^2\right) } \nonumber \\  &   \times \left( 8 (D+1) M_H^2 M_W^4 \left( M_H^2-4 M_Z^2\right) \right. \nonumber \\  &   +16 (D-1)M_W^6 \left( 4 M_Z^2-M_H^2\right) \nonumber \\  &   + \left. 4 M_W^2 \left( -4 (D+1) M_H^2 M_Z^4+8 (D-1) M_Z^6+30 M_H^4 M_Z^2-7 M_H^6\right) \right. \nonumber \\  &   +4 (D+1) M_H^4 M_Z^4-8 (D-1) M_H^2 M_Z^6 \nonumber \\  &   + \left. 4 M_H^2 m_n^2 \left( M_H^2-4 M_W^2\right) \left( M_H^2-4 M_Z^2\right) \right. \nonumber \\  &   +8m_n^4 \left( 4 M_W^2-M_H^2\right) \left( M_H^2-4 M_Z^2\right) -26 M_H^6 M_Z^2+6 M_H^8) \Biggr ] . \end{aligned}$$

## Appendix C Gravitational form factors

One-loop contributions to the GFFs of the Higgs boson have the form

$$\begin{aligned}  &   \theta _1 (t) = \frac{9 e^2 \left( 12 M_H^4+4 (D-3) t M_H^2-(D-2) t^2\right) M_Z^2 M_H^4 C_0\left( M_H^2,M_H^2,t,M_H^2,M_H^2,M_H^2\right) }{64 (D-2) \pi ^2 t \left( 4 M_H^2-t\right) M_W^2 \left( M_W^2-M_Z^2\right) } \nonumber \\  &   \quad - \frac{3 e^2 \left( 8 (D-5) M_H^6+4 \left( 2 D^2+3 D-5\right) t M_H^4+2 \left( D^2-12 D+14\right) t^2 M_H^2-(D-2)^2 t^3\right) M_Z^2 M_H^2 B_0\left( t,M_H^2,M_H^2\right) }{128 (D-2) (D-1) \pi ^2 t \left( M_H^2-t\right) \left( 4 M_H^2-t\right) M_W^2 \left( M_W^2-M_Z^2\right) }\nonumber \\  &   \quad + \frac{3 e^2 \left( -12 (D-3) M_H^4-(D-2) t M_H^2+(D-2) t^2\right) M_Z^2 M_H^2 B_0\left( M_H^2,M_H^2,M_H^2\right) }{64 (D-2) \pi ^2 t \left( 4 M_H^2-t\right) M_W^2 \left( M_W^2-M_Z^2\right) }\nonumber \\  &   \quad + \frac{3 e^2 \left( 2 (D-2) M_H^4+(2 D-3) t M_H^2-(D-1) t^2\right) M_Z^2 A_0\left( M_H^2\right) }{64 (D-1) \pi ^2 t \left( M_H^2-t\right) M_W^2 \left( M_W^2-M_Z^2\right) } \nonumber \\  &   \quad + \frac{e^2 m_n^2 M_Z^2 B_0\left( t,m_n^2,m_n^2\right) }{16 (D-2) (D-1) \pi ^2 t \left( M_H^2-t\right) \left( 4 M_H^2-t\right) M_W^2 \left( M_W^2-M_Z^2\right) }\nonumber \\  &   \quad \times \biggl [ 4 (D-1) M_H^6-8 (2 D-3) t M_H^4 \nonumber \\  &   \quad + (3 D-1) t^2 M_H^2-t^3+4 m_n^2 \left( 4 (D-3) M_H^4+2 (4 D-5) t M_H^2+(4-3 D) t^2\right) \biggr ] \nonumber \\  &   \quad + \frac{e^2 M_Z^2 B_0\left( t,M_W^2,M_W^2\right) }{64 (D-2) (D-1) \pi ^2 t \left( M_H^2-t\right) \left( 4 M_H^2-t\right) M_W^2 \left( M_W^2-M_Z^2\right) } \nonumber \\  &   \quad \times \biggl [ 8 (D-1) M_H^8-4 \left( 2 D^2-5 D+5\right) t M_H^6 \nonumber \\  &   \quad -2 \left( D^2-6 D+6\right) t^2 M_H^4+(D-2)^2 t^3 M_H^2\nonumber \\  &   \quad -8 \left( 4 \left( D^2-4 D+3\right) M_H^4+2 \left( 4 D^2-9 D+5\right) t M_H^2 \right. \nonumber \\  &   \quad + \left. \left( -3 D^2+7 D-4\right) t^2\right) M_W^4-2 \left( 16 (2 D-3)M_H^6 \right. \nonumber \\  &   \quad +4 \left( 2 D^3-18 D^2+31 D-16\right) t M_H^4 \nonumber \\  &   \quad + \left. 2 \left( D^3-9 D^2+35 D-26\right) t^2 M_H^2-\left( D^3-9 D^2+28 D-20\right) t^3\right) M_W^2 \biggr ] \nonumber \\  &   \quad + \frac{e^2 M_Z^2 B_0\left( t,M_Z^2,M_Z^2\right) }{128 (D-2) (D-1) \pi ^2 t \left( M_H^2-t\right) \left( 4 M_H^2-t\right) M_W^2 \left( M_W^2-M_Z^2\right) }\nonumber \\  &   \quad \times \biggl [ 8 (D-1) M_H^8-4 \left( 2 D^2-5 D+5\right) t M_H^6 \nonumber \\  &   \quad - 2 \left( D^2-6 D+6\right) t^2 M_H^4+(D-2)^2 t^3 M_H^2\nonumber \\  &   \quad -8 \left( 4 \left( D^2-4 D+3\right) M_H^4+2 \left( 4 D^2-9 D+5\right) t M_H^2 \right. \nonumber \\  &   \quad + \left. \left( -3 D^2+7 D-4\right) t^2\right) M_Z^4-2 \left( 16 (2 D-3) M_H^6\right. \nonumber \\  &   \quad +4 \left( 2 D^3-18 D^2+31 D-16\right) t M_H^4 \nonumber \\  &   \quad + \left. 2 \left( D^3-9 D^2+35 D-26\right) t^2 M_H^2-\left( D^3-9 D^2+28 D-20\right) t^3\right) M_Z^2 \biggr ] \nonumber \\  &   \quad + \frac{e^2 m_n^2 \left( -2 M_H^6+t M_H^4+8 m_n^4 \left( t-4 M_H^2\right) +m_n^2 \left( 16 M_H^4-6 t M_H^2+t^2\right) \right) M_Z^2 C_0\left( M_H^2,M_H^2,t,m_n^2,m_n^2,m_n^2\right) }{8 (D-2) \pi ^2 t \left( 4 M_H^2-t\right) M_W^2 \left( M_W^2-M_Z^2\right) }\nonumber \\  &   \quad + \frac{e^2 M_Z^2 C_0\left( M_H^2,M_H^2,t,M_W^2,M_W^2,M_W^2\right) }{32 (D-2) \pi ^2 t \left( 4 M_H^2-t\right) M_W^2 \left( M_W^2-M_Z^2\right) } \nonumber \\  &   \quad \times \biggl [ -4 M_H^8+4 (D-2) t M_H^6-(D-2) t^2 M_H^4 \nonumber \\  &   \quad + 16 (D-1) \left( 4 M_H^2-t\right) M_W^6-4 \left( 4 (D+3) M_H^4 \right. \nonumber \\  &   \quad -4 \left. \left( D^2-7 D+15\right) t M_H^2+\left( D^2-7 D+14\right) t^2\right) M_W^4 \nonumber \\  &   \quad + 2 \left( 16 M_H^6+(6-8 D) t M_H^4+6 (D-2) t^2 M_H^2-(D-2) t^3\right) M_W^2 \biggr ] \nonumber \\  &   \quad + \frac{e^2 M_Z^2 C_0\left( M_H^2,M_H^2,t,M_Z^2,M_Z^2,M_Z^2\right) }{64 (D-2) \pi ^2 t \left( 4 M_H^2-t\right) M_W^2 \left( M_W^2-M_Z^2\right) } \nonumber \\  &   \quad \times \biggl [ -4 M_H^8+4 (D-2) t M_H^6-(D-2) t^2 M_H^4 \nonumber \\  &   \quad +16 (D-1) \left( 4 M_H^2-t\right) M_Z^6-4 \left( 4 (D+3) M_H^4 \right. \nonumber \\  &   \quad -4\left. \left( D^2-7 D+15\right) t M_H^2+\left( D^2-7 D+14\right) t^2\right) M_Z^4 \nonumber \\  &   \quad + 2 \left( 16 M_H^6+(6-8 D) t M_H^4+6 (D-2) t^2 M_H^2-(D-2) t^3\right) M_Z^2 \biggr ] \nonumber \\  &   \quad - \frac{e^2 M_Z^2 }{64 \pi ^2 M_W^2 \left( 4 M_W^2-M_H^2\right) \left( M_W^2-M_Z^2\right) \left( 4 M_Z^2-M_H^2\right) M_H^2} \nonumber \\  &   \quad \times \biggl [ 3 M_H^8-13 M_Z^2 M_H^6+2 (D+1) M_Z^4 M_H^4 \nonumber \\  &   \quad - 4 (D-1) M_Z^6 M_H^2+4 (D+1) M_W^4 \left( M_H^2-4 M_Z^2\right) M_H^2 \nonumber \\  &   \quad +2 m_n^2 \left( M_H^2-4 M_W^2\right) \left( M_H^2-4 M_Z^2\right) M_H^2 \nonumber \\  &   \quad + 4 m_n^4 \left( 4 M_W^2-M_H^2\right) \left( M_H^2-4 M_Z^2\right) +8 (D-1) M_W^6 \left( 4 M_Z^2-M_H^2\right) \nonumber \\  &   \quad +2 M_W^2 \left( -7 M_H^6+30 M_Z^2 M_H^4-4 (D+1) M_Z^4 M_H^2+8 (D-1) M_Z^6\right) \biggr ] \nonumber \\  &   \quad + \frac{e^2 m_n^2 \left( -4 (D-2) M_H^4+(5-3 D) t M_H^2+(D-1) t^2\right) M_Z^2 A_0\left( m_n^2\right) }{16 (D-1) \pi ^2 t \left( M_H^2-t\right) M_W^2 \left( M_W^2-M_Z^2\right) M_H^2} \end{aligned}$$

C1 $$\begin{aligned}  &   \quad + \frac{e^2 M_Z^2 A_0\left( M_W^2\right) }{32 (D-1) \pi ^2 t \left( M_H^2-t\right) M_W^2 \left( 4 M_W^2-M_H^2\right) \left( M_W^2-M_Z^2\right) M_H^2}\nonumber \\  &   \quad \times \biggl [ -2 (D-2) M_H^8+t M_H^6-(D-1) t^2 M_H^4 \nonumber \\  &   \quad + 4 \left( 4 \left( D^2-3 D+2\right) M_H^4+\left( 3 D^2-8 D+5\right) t M_H^2 \right. \nonumber \\  &   \quad \left. -(D-1)^2 t^2\right) M_W^4-2 \left( 2 \left( D^2-5 D+6\right) M_H^6 \right. \nonumber \\  &   \quad + \left. \left( D^2-3 D+4\right) t M_H^4-2 (D-1) t^2 M_H^2\right) M_W^2 \biggr ] \nonumber \\  &   \quad + \frac{e^2 M_Z^2 A_0\left( M_Z^2\right) }{64 (D-1) \pi ^2 t \left( M_H^2-t\right) M_W^2 \left( M_W^2-M_Z^2\right) \left( 4 M_Z^2-M_H^2\right) M_H^2} \nonumber \\  &   \quad \times \biggl [ -2 (D-2) M_H^8+t M_H^6-(D-1) t^2 M_H^4 \nonumber \\  &   \quad + 4 \left( 4 \left( D^2-3 D+2\right) M_H^4+\left( 3 D^2-8 D+5\right) t M_H^2 \right. \nonumber \\  &   \quad \left. -(D-1)^2 t^2\right) M_Z^4-2 \left( 2 \left( D^2-5 D+6\right) M_H^6 \right. \nonumber \\  &   \quad + \left. \left( D^2-3 D+4\right) t M_H^4-2 (D-1) t^2 M_H^2\right) M_Z^2 \biggr ] \nonumber \\  &   \quad - \frac{e^2 \left( \left( 2 (D-2) t^2-32 (D-3) M_H^4\right) m_n^4+M_H^2 \left( 2 M_H^2-t\right) \left( 4 (D-3) M_H^2-(D-2) t\right) m_n^2\right) M_Z^2 B_0\left( M_H^2,m_n^2,m_n^2\right) }{32 (D-2) \pi ^2 t \left( 4 M_H^2-t\right) M_W^2 \left( M_W^2-M_Z^2\right) M_H^2} \nonumber \\  &   \quad - \frac{e^2 M_Z^2 B_0\left( M_H^2,M_W^2,M_W^2\right) }{32 (D-2) \pi ^2 t \left( 4 M_H^2-t\right) M_W^2 \left( 4 M_W^2-M_H^2\right) \left( M_W^2-M_Z^2\right) M_H^2} \nonumber \\  &   \quad \times \biggl [ \left( (D-2) t-4 (D-3) M_H^2\right) M_H^8 \nonumber \\  &   \quad + 4 \left( 16 \left( D^2-4 D+3\right) M_H^4-\left( D^2-3 D+2\right) t^2\right) M_W^6 \nonumber \\  &   \quad -4 \left( 4 \left( D^2-9\right) M_H^6-\left( D^2+13 D-46\right) t M_H^4 \right. \nonumber \\  &   \quad + \left. 3 (D-2) t^2 M_H^2\right) M_W^4+\left( 32 (D-3) M_H^8 \right. \nonumber \\  &   \quad \left. +4 (14-5 D) t M_H^6+3 (D-2) t^2 M_H^4\right) M_W^2 \biggr ] \nonumber \\  &   \quad - \frac{e^2 M_Z^2 B_0\left( M_H^2,M_Z^2,M_Z^2\right) }{64 (D-2) \pi ^2 t \left( 4 M_H^2-t\right) M_W^2 \left( M_W^2-M_Z^2\right) \left( 4 M_Z^2-M_H^2\right) M_H^2} \biggl [ \left( (D-2) t-4 (D-3) M_H^2\right) M_H^8 \nonumber \\  &   \quad + 4 \left( 16 \left( D^2-4 D+3\right) M_H^4-\left( D^2-3 D+2\right) t^2\right) M_Z^6-4 \left( 4 \left( D^2-9\right) M_H^6-\left( D^2+13 D-46\right) t M_H^4 \right. \nonumber \\  &   \quad + \left. 3 (D-2) t^2 M_H^2\right) M_Z^4+\left( 32 (D-3) M_H^8 \right. \nonumber \\  &   \quad \left. +4 (14-5 D) t M_H^6+3 (D-2) t^2 M_H^4\right) M_Z^2 \biggr ]. \end{aligned}$$

C2 $$\begin{aligned}  &   \theta _2 (t) = \frac{9 e^2 \left( 4 (D-5) M_H^4-4 (D-3) t M_H^2+(D-2) t^2\right) M_Z^2 C_0\left( M_H^2,M_H^2,t,M_H^2,M_H^2,M_H^2\right) M_H^4}{64 (D-2) \pi ^2 \left( t-4 M_H^2\right) {}^2 M_W^2 \left( M_W^2-M_Z^2\right) }\nonumber \\  &   \quad + \frac{3 e^2 \left( 4 (2 D-7) M_H^4-(D-2) t M_H^2-(D-2) t^2\right) M_Z^2 B_0\left( M_H^2,M_H^2,M_H^2\right) M_H^2}{64 (D-2) \pi ^2 \left( t-4 M_H^2\right) {}^2 M_W^2 \left( M_W^2-M_Z^2\right) }\nonumber \\  &   \quad +\frac{3 e^2 M_Z^2 A_0\left( M_H^2\right) }{64 \pi ^2 M_W^2 \left( M_W^2-M_Z^2\right) } \nonumber \\  &   \quad - \frac{e^2 m_n^2 \left( 4 (D-1) M_H^4-4 t M_H^2+t^2-8 (D-1) m_n^2 \left( 2 M_H^2-t\right) \right) M_Z^2 B_0\left( t,m_n^2,m_n^2\right) }{16 (D-2) \pi ^2 \left( t-4 M_H^2\right) {}^2 M_W^2 \left( M_W^2-M_Z^2\right) } \nonumber \\  &   \quad - \frac{e^2 M_Z^2 B_0\left( t,M_W^2,M_W^2\right) }{16 (D-2) \pi ^2 \left( t-4 M_H^2\right) {}^2 M_W^2 \left( M_W^2-M_Z^2\right) } \nonumber \\  &   \quad \times \biggl [ (D-1) \left( 2 M_H^2-t\right) M_H^4+4 (D-1)^2 \left( 2 M_H^2-t\right) M_W^4 \nonumber \\  &   \quad - 4 \left( 2 (D-1) M_H^4+(D-5) t M_H^2+t^2\right) M_W^2 \biggr ] \nonumber \\  &   \quad -\frac{9 (D-1) e^2 \left( 2 M_H^2-t\right) M_Z^2 B_0\left( t,M_H^2,M_H^2\right) M_H^4}{32 (D-2) \pi ^2 \left( t-4 M_H^2\right) {}^2 M_W^2 \left( M_W^2-M_Z^2\right) } \nonumber \\  &   \quad - \frac{e^2 M_Z^2 B_0\left( t,M_Z^2,M_Z^2\right) }{32 (D-2) \pi ^2 \left( t-4 M_H^2\right) {}^2 M_W^2 \left( M_W^2-M_Z^2\right) }\nonumber \\  &   \quad \times \biggl [ (D-1) \left( 2 M_H^2-t\right) M_H^4+4 (D-1)^2 \left( 2 M_H^2-t\right) M_Z^4 \nonumber \\  &   \quad - 4 \left( 2 (D-1) M_H^4+(D-5) t M_H^2+t^2\right) M_Z^2 \biggr ] \nonumber \\  &   \quad + \frac{e^2 m_n^2 M_Z^2 C_0\left( M_H^2,M_H^2,t,m_n^2,m_n^2,m_n^2\right) }{8 (D-2) \pi ^2 \left( t-4 M_H^2\right) {}^2 M_W^2 \left( M_W^2-M_Z^2\right) }\nonumber \\  &   \quad \times \biggl [ 8 \left( 4 M_H^2-t\right) m_n^4+\left( t \left( 3 t-10 M_H^2\right) -2 D \left( t-2 M_H^2\right) {}^2\right) m_n^2 \nonumber \\  &   \quad + (D-1) M_H^4 \left( 2 M_H^2-t\right) \biggr ] \nonumber \\  &   \quad + \frac{e^2 M_Z^2 C_0\left( M_H^2,M_H^2,t,M_W^2,M_W^2,M_W^2\right) }{32 (D-2) \pi ^2 \left( t-4 M_H^2\right) {}^2 M_W^2 \left( M_W^2-M_Z^2\right) }\nonumber \\  &   \quad \times \biggl [ 4 (D-1) M_H^8-4 (D-2) t M_H^6+(D-2) t^2 M_H^4 \nonumber \\  &   \quad - 16 (D-1) \left( 4 M_H^2-t\right) M_W^6+4 \left( 4 \left( D^2-2 D+5\right) M_H^4\right. \nonumber \\  &   \quad \left. -4\left( D^2-3 D+7\right) t M_H^2+\left( D^2-3 D+6\right) t^2\right) M_W^4 \nonumber \\  &   \quad - 2 \left( 2 t \left( 11 M_H^4-6 t M_H^2+t^2\right) +D \left( 8 M_H^6-16 t M_H^4+6 t^2 M_H^2-t^3\right) \right) M_W^2 \biggr ] \nonumber \\  &   \quad + \frac{e^2 M_Z^2 C_0\left( M_H^2,M_H^2,t,M_Z^2,M_Z^2,M_Z^2\right) }{64 (D-2) \pi ^2 \left( t-4 M_H^2\right) {}^2 M_W^2 \left( M_W^2-M_Z^2\right) }\nonumber \\  &   \quad \times \biggl [ 4 (D-1) M_H^8-4 (D-2) t M_H^6+(D-2) t^2 M_H^4 \nonumber \\  &   \quad - 16 (D-1) \left( 4 M_H^2-t\right) M_Z^6+4 \left( 4 \left( D^2-2 D+5\right) M_H^4\right. \nonumber \\  &   \quad \left. -4\left( D^2-3 D+7\right) t M_H^2+\left( D^2-3 D+6\right) t^2\right) M_Z^4 \nonumber \\  &   \quad - 2 \left( 2 t \left( 11 M_H^4-6 t M_H^2+t^2\right) +D \left( 8 M_H^6-16 t M_H^4+6 t^2 M_H^2-t^3\right) \right) M_Z^2 \biggr ] \nonumber \\  &   \quad - \frac{e^2 M_Z^2 }{64 \pi ^2 M_W^2 \left( 4 M_W^2-M_H^2\right) \left( M_W^2-M_Z^2\right) \left( 4 M_Z^2-M_H^2\right) M_H^2} \nonumber \\  &   \quad \times \biggl [ 3 M_H^8-13 M_Z^2 M_H^6+2 (D+1) M_Z^4 M_H^4 \nonumber \\  &   \quad - 4 (D-1) M_Z^6 M_H^2+4 (D+1) M_W^4 \left( M_H^2-4 M_Z^2\right) M_H^2 \nonumber \\  &   \quad +2 m_n^2 \left( M_H^2-4 M_W^2\right) \left( M_H^2-4 M_Z^2\right) M_H^2 \nonumber \\  &   \quad + 4 m_n^4 \left( 4 M_W^2-M_H^2\right) \left( M_H^2-4 M_Z^2\right) +8 (D-1) M_W^6 \left( 4 M_Z^2-M_H^2\right) \nonumber \\  &   \quad + 2 M_W^2 \left( -7 M_H^6+30 M_Z^2 M_H^4-4 (D+1) M_Z^4 M_H^2\right. \nonumber \\  &   \quad \left. +8 (D-1) M_Z^6\right) \biggr ] \nonumber \\  &   \quad - \frac{e^2 m_n^2 M_Z^2 A_0\left( m_n^2\right) }{16 \pi ^2 M_W^2 \left( M_W^2-M_Z^2\right) M_H^2}\nonumber \\  &   \quad +\frac{e^2 \left( M_H^4-4 M_W^2 M_H^2+4 (D-1) M_W^4\right) M_Z^2 A_0\left( M_W^2\right) }{32 \pi ^2 M_W^2 \left( 4 M_W^2-M_H^2\right) \left( M_W^2-M_Z^2\right) M_H^2} \nonumber \\  &   \quad + \frac{e^2 m_n^2 M_Z^2 B_0\left( M_H^2,m_n^2,m_n^2\right) }{32 (D-2) \pi ^2 \left( t-4 M_H^2\right) {}^2 M_W^2 \left( M_W^2-M_Z^2\right) M_H^2} \nonumber \\  &   \quad \times \biggl [ 8 (2 D-5) M_H^6+2 (8-3 D) t M_H^4+(D-2) t^2 M_H^2 \nonumber \\  &   \quad + m_n^2 \left( -32 (D-3) M_H^4+8 (D-2) t M_H^2+2 (D-2) t^2\right) \biggr ] \nonumber \\  &   \quad + \frac{e^2 M_Z^2 B_0\left( M_H^2,M_W^2,M_W^2\right) }{32 (D-2) \pi ^2 \left( t-4 M_H^2\right) {}^2 M_W^2 \left( 4 M_W^2-M_H^2\right) \left( M_W^2-M_Z^2\right) M_H^2} \biggl [ \left( (20-8 D) M_H^2+3 (D-2) t\right) M_H^8 \nonumber \\  &   \quad + 4 \left( 16 \left( D^2-4 D+3\right) M_H^4-4 \left( D^2-3 D+2\right) t M_H^2-\left( D^2-3 D+2\right) t^2\right) M_W^6 -4 \left( 4 \left( 2 D^2-3 D-7\right) M_H^6 \right. \nonumber \\  &   \quad + \left. \left( -3 D^2+5 D+18\right) t M_H^4+3 (D-2) t^2 M_H^2\right) M_W^4\nonumber \\  &   \quad +\left( 16 (3 D-8) M_H^8-16 (D-3) t M_H^6+3 (D-2) t^2 M_H^4\right) M_W^2 \biggr ] \nonumber \\  &   \quad + \frac{e^2 M_Z^2 B_0\left( M_H^2,M_Z^2,M_Z^2\right) }{64 (D-2) \pi ^2 \left( t-4 M_H^2\right) {}^2 M_W^2 \left( M_W^2-M_Z^2\right) \left( 4 M_Z^2-M_H^2\right) M_H^2} \nonumber \\  &   \quad \times \biggl [ \left( (20-8 D) M_H^2+3 (D-2) t\right) M_H^8 \nonumber \\  &   \quad + 4 \left( 16 \left( D^2-4 D+3\right) M_H^4-4\left( D^2-3 D+2\right) t M_H^2\right. \nonumber \\  &   \quad \left. -\left( D^2-3 D+2\right) t^2\right) M_Z^6 - 4 \left( 4 \left( 2 D^2-3 D-7\right) M_H^6 \left( 18-3 D^2+5 D\right) t M_H^4 + 3 (D-2) t^2 M_H^2\right) M_Z^4 \nonumber \\  &   \quad +\left( 16 (3 D-8) M_H^8-16 (D-3) t M_H^6+3 (D-2) t^2 M_H^4\right) M_Z^2 \biggr ] \nonumber \\  &   \quad + \frac{e^2 M_Z^2 \left( M_H^4-4 M_Z^2 M_H^2+4 (D-1) M_Z^4\right) A_0\left( M_Z^2\right) }{64 \pi ^2 M_W^2 \left( M_W^2-M_Z^2\right) \left( 4 M_Z^2-M_H^2\right) M_H^2}. \end{aligned}$$

## Code Availability Statement

Code/software will be made available on reasonable request. [Authors’ comment: The code/software generated during and/or analysed during the current study is available from the corresponding author on reasonable request].
